# Milk Fat Globule Membrane Supplementation in Children: Systematic Review with Meta-Analysis

**DOI:** 10.3390/nu13030714

**Published:** 2021-02-24

**Authors:** Dominika Ambrożej, Karolina Dumycz, Piotr Dziechciarz, Marek Ruszczyński

**Affiliations:** 1 Department of Pediatric Pneumonology and Allergy, Medical University of Warsaw, 02-091 Warsaw, Poland; dominika.ambrozej@wum.edu.pl (D.A.); karolina.dumycz@wum.edu.pl (K.D.); 2 Doctoral School, Medical University of Warsaw, 02-091 Warsaw, Poland; 3 Department of Pediatrics, Medical University of Warsaw, 02-091 Warsaw, Poland; piotr.dziechciarz@wum.edu.pl

**Keywords:** milk fat globule membrane, MFGM, feeding, infant formula, children

## Abstract

(1) Background: Milk fat globule membrane (MFGM), composing fat droplets responsible for lipid transport in breast milk, has been shown to possess immunological and antimicrobial effects. Standard formulas (SF) are devoid of MFGMs during the production process. The study’s aim was to evaluate the safety and benefits of MFGMs supplementation in children. (2) Methods: We searched four databases for randomized controlled trials evaluating the supplementation of MFGMs in children. Growth parameters were chosen as the primary outcome. (3) Results: Twenty-four publications of seventeen studies were included. Meta-analyses assessing the primary outcomes at the age of 4 months included four studies (814 children) comparing the MFGM-supplemented formulas and SF, and two trials (549 children) comparing the MFGM-supplemented formulas and breastfeeding. The primary outcomes were non-inferior in all the experimental MFGM formulas compared to SF, or even represented more similar results to breastfed infants. The promising effects, including a lower incidence of acute otitis media and improved cognitive development, cannot be firmly confirmed due to the small amount of existing evidence. No significant adverse effects were reported in any of the assessed products. (4) Conclusions: The available data signaled beneficial effects and a good safety profile, requiring future research with well-designed trials.

## 1. Introduction

Breastfeeding is the most effective and natural method of feeding to provide infants with valuable nutrients, immunomodulatory factors, and energy [1]. It has been long reported to have various protective effects against allergies, asthma, infections, and immune-mediated diseases in children [2,3]. The exact composition of human breast milk depends on various maternal factors and matches specific infant demands. The largest energy source in human milk forms lipids, which contribute approximately 40–55% of the total energy [4]. Lipids are secreted into breast milk by an exclusive mammary gland mechanism and are composed of fat droplets enclosed with a tri-layer membrane [5]. The core lipid consists mainly of triglycerides (98%), whereas the membrane, called milk fat globule membrane (MFGM), comprises a monolayer of polar lipids and a lipid bilayer. MFGM consists of phospholipids such as sphingomyelins, phosphatidylcholines, gangliosides, and different proteins, including lactoferrin or mucins [6].

An increasing body of evidence indicates that MFGM can exert a beneficial effect on immune functions, central nervous system development, and metabolism [7]. Standard formulas are devoid of bovine MFGMs due to the production process and are replaced by vegetable oils. This phenomenon may account for the differences observed between formula-fed and breastfed infants. Therefore, in recent years, efforts have been made to create standard formulas closely imitating human milk by adding bovine MFGM to the composition.

The MFGMs-supplemented formulas are attracting an increasing interest due to the reports on their favorable effectiveness on infants’ growth, risk of infections, cognitive development, microbiome, and metabolism in comparison to standard formulas and/or breastfeeding [8].

The purpose of this systematic review was to evaluate the safety and clinical benefits of the MFGMs supplementation in children available from randomized controlled trials.

## 2. Materials and Methods

### 2.1. Search Strategy

The study followed the Preferred Reporting Items for Systematic Reviews and Meta-Analyses (PRISMA) guidelines and was registered in the National Institute for Health Research’s PROSPERO (CRD42020138870) [9].

We performed a systematic search and retrieved studies from PubMed, Web of Science, Embase, and the Cochrane’s Library databases from inception to 20 October 2019. Additionally, the hand-search was conducted. We have repeated the same search on 18 July 2020 for new data through all previously used databases to keep our systematic review up to date. The search terms with the filter validated in the Cochrane Handbook for randomized controlled trials can be found in the Supplementary Materials (Table S1).

### 2.2. Study Selection

All extracted papers were imported into EndNote® reference manager Version x8 (Clarivate Analytics; 2016). Two reviewers working independently (D.A., M.R.) screened at first the titles with abstracts and next assessed the potentially suitable full texts. Uncertainty about the eligibility of studies for the review was resolved through discussion at each stage with a third reviewer (P.D.). The final group of selected studies met the inclusion and exclusion criteria pointed out herein.

### 2.3. Inclusion Criteria

Original studies were accepted only if they (1) were published in English; (2) were performed on human beings aged less than 18 years old; (3) introduced MFGMs oral supplementation performed in any available form; (4) involved any of the following control groups: placebo or milk formula without supplementation; and (5) have been performed as randomized controlled trials (RCTs).

### 2.4. Exclusion Criteria

We excluded (1) duplicate publications and (2) multiple publications of the same trial, (3) conference abstracts, (4) study protocols, (5) nonhuman studies, and (6) trials in which the MFGMs were administered during prenatal development.

### 2.5. Data Extraction

Data were independently extracted by three reviewers (D.A., K.D., and M.R.) using a structured data collection form, collating relevant study details such as date of publication, country of origin, study design, population of the study, intervention and comparison, measurement points, results, and confounding factors. Actions were taken to contact corresponding authors when additional clarification was required. Data were extracted from all selected articles, regardless of whether the results were positive or negative, to present a fair and unbiased account of the available published evidence.

### 2.6. Risk of Bias Assessment

Three independent reviewers (D.A., K.D., and M.R.) assessed the risk of bias, without being blinded to the authors or journal, in each of the included studies. A revised Cochrane risk-of-bias tool for randomized trials was used (RoB 2) [10], Figure S1. Encountered discrepancies were resolved through discussion of all the reviewers.

The RoB 2 tool is structured into five bias domains, which enables judging the randomization process, deviations from intended interventions, any missing outcome data, measurement of the outcome, and selection of the reported result.

The judgments for each domain were to choose between “low risk of bias”, “some concerns”, or “high risk of bias”. In conclusion, the overall bias was determined by reflecting the individual marks.

### 2.7. Data Synthesis

Required data were extracted from included studies to a predesigned Excel sheet in Microsoft Excel. Review Manager (RevMan) v5.3 software from the Cochrane Collaboration (London, UK) was used to perform meta-analyses, generate forest plots, and calculate the I^2^ statistic. The data were pooled on baseline characteristics and outcomes using the meta-analysis approach. In the studies that reported continuous variables as means, mean difference (MD) was calculated. Heterogeneity was assessed using the I^2^ statistic defined by the Cochrane Handbook for Systematic Reviews. Either Chi^2^ test *p* < 0.10 or I^2^ value > 50% indicated substantial heterogeneity.

Growth parameters such as weight, length/height, and head circumference were chosen as the primary outcome of our systematic review.

The secondary outcomes assessed in the review consisted of any additional outcomes reported in the included trials such as validated psychomotor development scales, any side effects, oral or gut microbiota shifts if reported, as well as any potential benefit on the infection/fever prevalence in children.

## 3. Results

### 3.1. Description of the Studies

**Figure 1:**
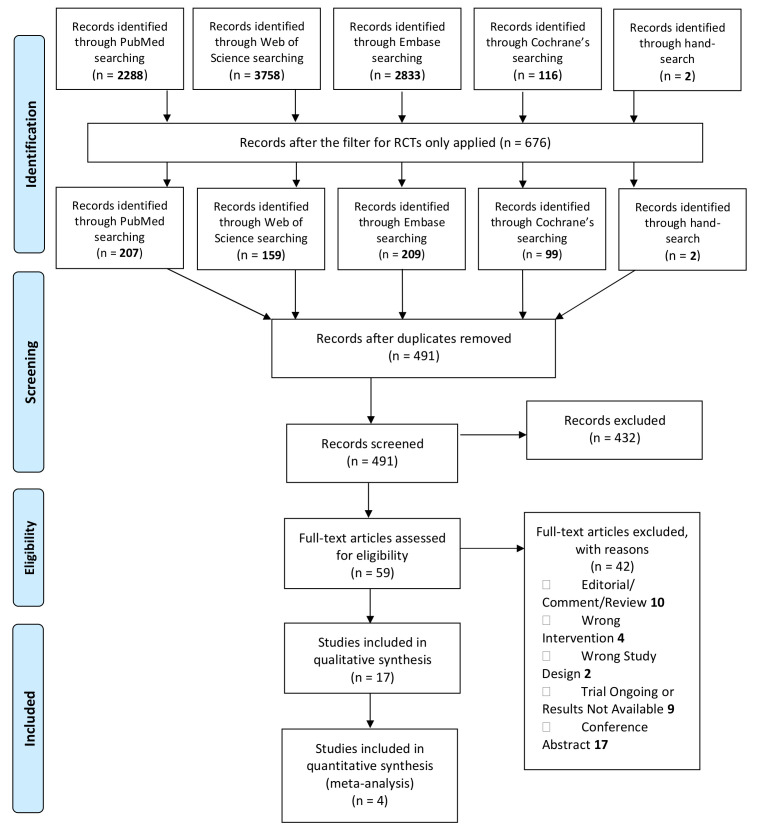
The Preferred Reporting Items for Systematic Reviews and Meta-Analyses (PRISMA) flow diagram.

Our initial search yielded 676 randomized controlled trials; after removing duplicates (*n* = 185), we identified 491 unique citations. The PRISMA flow chart is shown in Figure 1. The screening of titles and abstracts excluded 432 articles, rendering 59 trials to full-text assessment. Finally, 17 publications were included in our systematic review studies (Table 1). Data from three RCTs were eligible for the meta-analysis comparing anthropometric measurements in experimental vs. standard formula-fed infants [11,12,13], and two RCTs were eligible for meta-analysis comparing the same measurements in experimental formula vs. breast-fed infants [11,13].

All 10 clinical trials, including 3575 patients, were published in full text in peer-reviewed journals. Seven publications reported results from one clinical trial [11,14,15,16,17,18,19], and two publications reported results of another clinical trial [20,21]. Included trials were conducted in different parts of the world—five trials were conducted in Europe (Sweden, the Netherlands, Belgium, France, Italy, Spain) [11,12,21,22,23], one trial was conducted in South America (Peru) [24], and five trials were conducted in Asia (China, Indonesia, India, Singapore) [13,22,25,26,27]. Three of the trials were multicenter [12,22,23].

**Figure 2:**
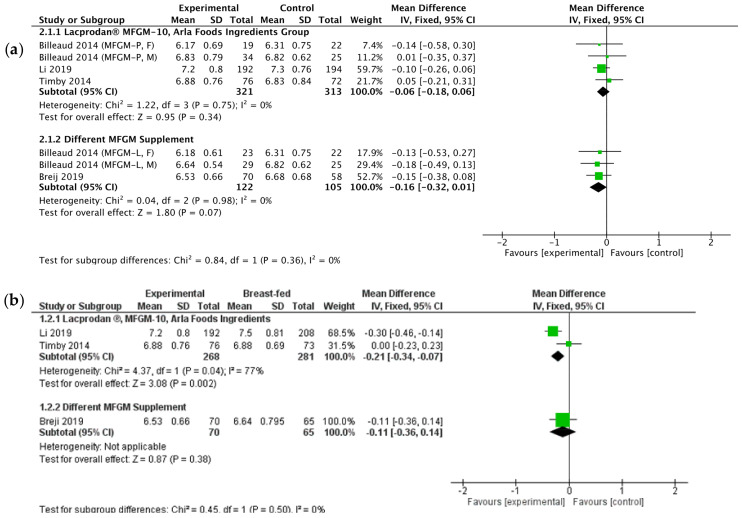

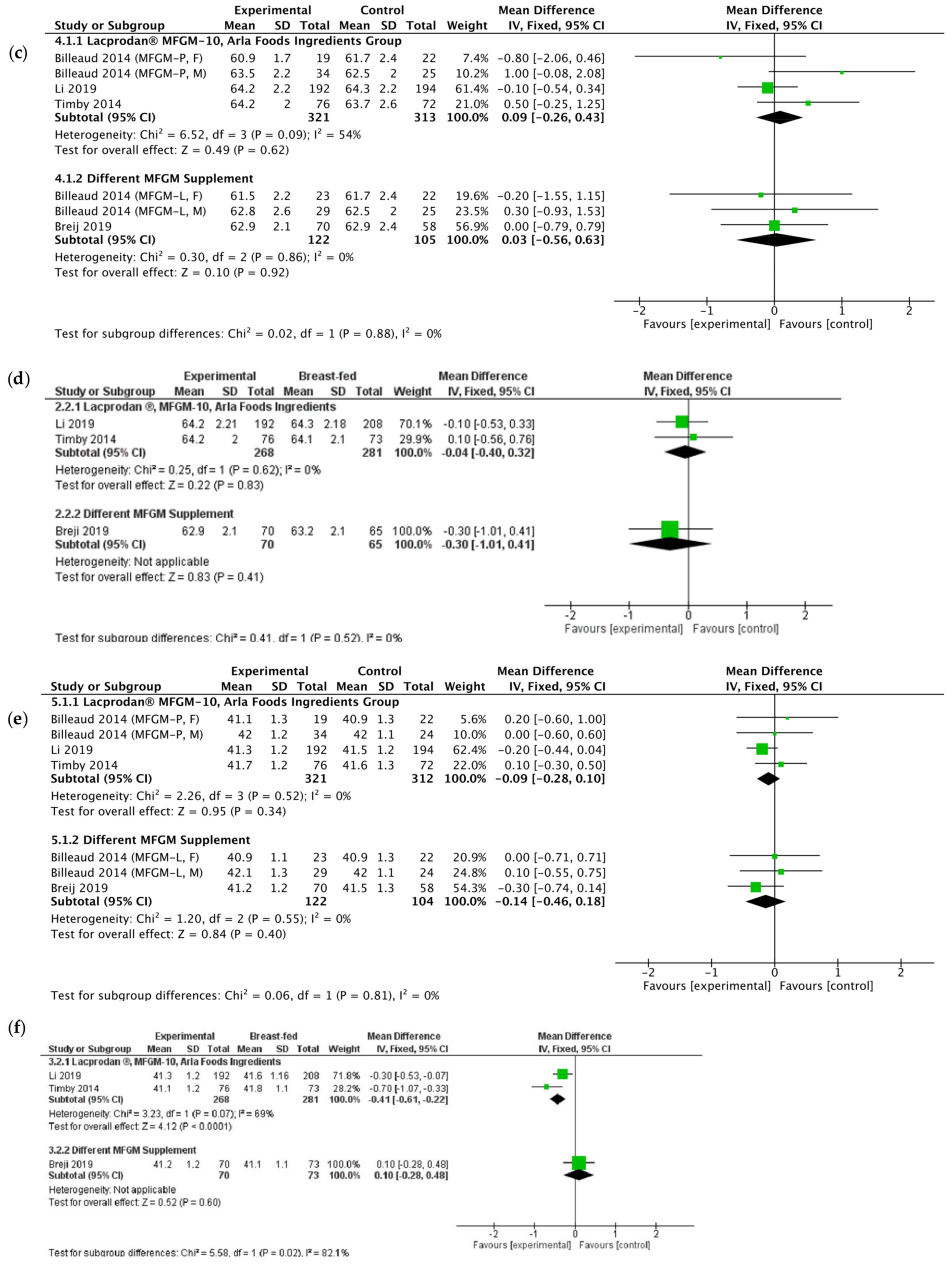
The meta-analyses of growth parameters measured at 4 months of age between children fed with standard formula or breastfed and formulas supplemented with milk fat globule membrane (MFGM): (**a**) Weight comparing the experimental formulas (including MFGM) with the standard formulas; (**b**) Weight comparing the experimental formulas (including MFGM) with breastfeeding; (**c**) Length comparing the experimental formulas (including MFGM) with the standard formulas; (**d**) Length comparing the experimental formulas (including MFGM) with breastfeeding; (**e**) Head circumference comparing the experimental formulas (including MFGM) with the standard formulas; (**f**) Head circumference comparing the experimental formulas (including MFGM) with breastfeeding.

In most of the included trials, the overall risk of bias was considered as low [11,12,13] or only with some concerns [22,24,26] (Figure 2). However, two of the studies were judged as with a high risk of bias [20,23]. Their methodological concerns included poor data availability on randomization and blinding process, deviations from the intended intervention, and potentially missing outcome data, as well as some discrepancies in reporting results. Notably, a few papers did not present the results according the intention to treat (ITT) analysis, which may have led to the increased bias [20,23,25,27]. Another potential confounder was the lack of the beforehand published trial protocol in three cases [23,24,25].

### 3.2. Populations

Most of the studies recruited participants below 2 months of age at inclusion. In the three remaining trials, older children were included (Table 1) [23,24,26].

### 3.3. Interventions

Clinical trials were heterogenous concerning applied MFGM-containing formula in the intervention groups. In one clinical trial, two different MFGM-containing formulas were used—lipid-rich MFGM (MFGM-L) (Anmum Infacare, Fonterra) and protein-rich MFGM (MFGM-P) (Lacprodan MFGM-10, Arla Food Ingredients) [12]. In four clinical trials, the latter formula was applied [11,13,24,27]; however, in one of them, it was applied along with the addition of bovine lactoferrin [27]. In the remaining studies, various types of MFGM-containing experimental formulas were used (Table 1) [20,23,25,26]. The duration of the intervention varied among the studies from approximately 3 months through 4, 6, to 18 months.

### 3.4. Outcomes

The outcomes presented in the trials were highly heterogenous in terms of the methods used to evaluate specific outcomes and time points of the assessment. In most of the trials’ anthropometric measurements (weight, length, head circumference, daily weight gain), cognitive development or infection prevalence were employed as the primary outcomes. Additional outcomes evaluated in the studies included adverse events, hematological and biochemical assessment, and oral and fecal microbiota.

Due to the heterogeneity of outcomes, most of the data were analyzed descriptively. Solely data on weight, length, and head circumference at four months of age were eligible for meta-analysis.

#### 3.4.1. Anthropometric Measurements

- *Experimental formula compared to a standard formula*

Data from the studies eligible for meta-analysis showed no differences in mean weight, length, and head circumference at the age of four months (Figure 2a,c,e) [11,12,13]. Furthermore, studies that were not included in the meta-analyses showed no differences in mean weight, length, head circumference, and mean weight gain [12,25,27]. Dissimilarities were also not shown with respect of weight-for-age, length-for-age, and weight-for-length z-scores [20,25].

- *Experimental Formula Compared to Breastfeeding*

The conducted meta-analyses indicated that at the age of four months, children from the group fed with experimental formula (EF) had slightly lower mean body weight and head circumference compared to the breastfed (BF) infants (Figure 2b,f). Body length did not differ between the groups (Figure 2d) [11,13].

Moreover, the comparison between BF and formula-fed (both EF + standard formula, SF) infants by Timby et al. revealed the significant interactions for z-scores for the weight (*p* = 0.025) and length (*p* = 0.003) [11]. On the other hand, Nieto-Ruiz et al. showed no statistically significant differences in mean weight, growth, daily weight gain, and no differences in several z-scores [20].

In the research conducted by Billeaud et al., one control group of standard formula-fed infants served as a comparison to infants fed with MFGM-P and MFGM-L formulas (F—females, M—males, MFGM-P—standard formula enriched with a protein-rich MFGM fraction, MFGM-L—standard formula enriched with a lipid-rich MFGM fraction).

#### 3.4.2. Psychomotor Development 

According to the results of the evaluation of 6-month-old infants using the Griffiths Mental Development Scale (GMDS) by Gurnida et al., there was a significant increase in scores for Hand and Eye Coordination (*p* = 0.006), Performance (*p* < 0.001), and for General IQ (*p* = 0.041) in the EF group. However, no significant difference was found between the EF and SF group for the Griffith Locomotor, Personal–Social, and Hearing and Speech scores. Remarkably, all scores in this group were similar to those of the BF group [25]. In two trials, infants’ psychomotor development was evaluated at 12 months of age using the Bayley Scales of Infant and Toddler Development (Baley-III) scale [11,27]. Timby et al. reported significantly higher scores of EF compared to SF groups in the cognitive domain (mean difference −4.0, 95%CI (−6.9498 to −1.0502), *p* = 0.008), but there were no differences compared to the BF group (*p* = 0.35). Moreover, BF infants performed significantly better in the verbal domain than both EF (mean difference −4.1, 95% CI (−7.6134 to −0.5866), *p* = 0.029) and SF infants (mean difference −4.2, 95% CI (−7.5807 to −0.8193), *p* = 0.012). No differences were reported in respect of the motor domain between any groups [11]. According to Li et al. EF-fed infants presented higher scores in the cognitive, language, and motor domains (*p* < 0.01; *p* < 0.01; *p* < 0.01) at 12 months of age. Differences did not persist until 18 months of age [27]. Nieto-Ruiz et al. indicated that children who had consumed EF during the first 18 months of life presented higher scores in the use of language (mean difference −5.86, 95% CI (−10.6048 to −1.1152) *p* = 0.033) and spontaneous oral expression (mean difference −0.150, 95% CI (−0.2998 to −0.0002) *p* = 0.024) than SF children according to the Oral Language Task of Navarra-Revised (PLON-R) test at the age of four [21]. Notably, Veereman et al. reported fewer behavioral problems assessed by parents using the Achenbach System of Empirically Based Assessment (ASEBA) questionnaire in children in the experimental group [23].

#### 3.4.3. Risk of Infections

The impact of applied formulas or breastfeeding on the prevalence of infections was evaluated in two manners in the included studies—either as a secondary endpoint or as an adverse event. Timby et al. reported a lower incidence of acute otitis media in the EF group (MFGM-P) compared to the SF group (*p* = 0.034) as well as the incidence of antipyretic use (*p* = 0.021) [15]. Likewise, Li et al. showed a lower incidence of respiratory tract infections and diarrhea in the EF group compared to the SF group [27]. According to Li et al. and Gurnida et al., MFGM-formula-fed and SF-fed infants did not differ significantly with respect to various infections [13,25]. By contrast, Breji et al. reported a higher occurrence of diarrhea in the EF group compared to the SF group (13.4% vs. 7.8%), though without statistical significance [22]. Furthermore, Billeaud et al. showed a slight difference in the prevalence of infections between infants in experimental and control groups [12]. Children in the MFGM-P group presented a significantly higher rate of respiratory tract infection symptoms reported by parents after 2 months of the beginning of the study. There were no differences in the infection rate between MFGM formula-fed infants and BF infants (except for one MFGM-P group). Bovine MFGM and phospholipids added to chocolate milk in pre-school children showed a reduction of days with fever compared to the control group (*p* < 0.028); however, there was no significant influence on the incidence of diarrhea, constipation, cough, doctors’ visits, and days of school absence [23].

#### 3.4.4. Prevention of Diarrhea in Developing Countries

Zavaleta et al. demonstrated a reduction of the number of bloody diarrhea episodes in the EF group as compared to the SF group (*p* = 0.025), although no significant effect on the longitudinal prevalence of diarrhea, duration of diarrhea episodes, and incidence of severe diarrhea episodes were reported in Peruvian infants. Furthermore, no differences in the pathogenesis of diarrhea were found (infection with different Escherichia coli serotypes, Campylobacter jejuni, Giardia lamblia, Blastocystis hominis, Rotavirus) [24]. Poppitt et al. reported no differences in the number, duration, and severity of all-cause diarrhea episodes between the EF and SF groups, however, the duration of rotavirus diarrhea was lower in the EF group (*p* = 0.03) throughout the intervention period [26].

#### 3.4.5. Metabolic Effects

Metabolic effects of several formulas were extensively evaluated in the cohort of children primarily described by Timby et al [11]. The first analysis revealed that EF and SF infants presented higher growth velocity than BF with concomitant higher plasma insulin and blood urea nitrogen level at six months of age [11]. Further analysis of lipidomics showed higher levels of triglycerides and cholesterol in serum, as well as higher low-density lipoprotein cholesterol: high-density lipoprotein cholesterol (LDL:HDL) ratios and leptin:fat mass ratios in BF infants compared to SF. The EF group also had a higher total serum cholesterol concentration than the SF group, reaching the level of the BF group [14]. Grip et al. indicated significant differences in the serum, plasma, and erythrocyte membrane concentrations of sphingomyelins, phosphatidylocholines, and ceramides at 4 and 6 months of age between infants from the EF and SF groups [17]. Moreover, He et al. demonstrated that the formula-fed infants presented a preference for protein metabolism, which was expressed by the higher serum levels of amino-acid catabolism products and low efficiency of amino-acid clearance. In contrast, BF infants had higher levels of fatty acid oxidation products, which account for fat metabolism preference. Interestingly, the EF group was characterized by higher levels of fat metabolism products than SF infants, which may bridge the gap between the EF and BF infants [18]. Metabolic differences were visible only during the exclusive feeding period and did not remain at 12 months of age [14,17,18].

Gurnida et al. revealed higher ganglioside serum concentrations in the EF group than SF (*p* < 0.01) but no significant differences between EF and BF infants at 6 months of age [25].

On the other hand, Billeaud et al. did not show any differences in plasma and red blood cell membrane levels of phospholipids, cardiolipin, cholesterol, insulin-like growth factor 1 (IGF-1), leptin nor IGF BP3 between EF and SF infants. However, the level of C-peptide was lower in both EF groups compared to SF (*p* = 0.03) [12].

#### 3.4.6. Microbiome

The oral and fecal microbiome was evaluated in infants from the cohort primarily described by Timby et al. Assessment of oral microbiota using the bacterial 16S rRNA sequencing method revealed significantly higher microbial richness in formula-fed (EF + SF) compared to BF infants at 4 months of age. Moreover, infants fed with EF had a significantly lower level of *Moraxella catarrhalis* compared to the SF-fed group, which may be associated with fewer episodes of acute otitis media in the EF group [16]. Yet, He showed that BF infants had a more heterogenous fecal microbiome but only during the exclusive formula feeding period. After the introduction of weaning food with either EF, SF, or BF, no significant differences in fecal microbiome were observed [19].

#### 3.4.7. Adverse Events

Treatment-emergent adverse events were reported as respiratory tract and gastrointestinal infections, skin diseases, and formula intolerance. Risk of respiratory tract and gastrointestinal infections was described in the Section 3.4.4, since in some of the trials, they were assessed in the character of primary outcomes. None of the studies raised the concerns of MFGM-supplemented formulas. No adverse events in constipation and skin diseases including eczema were reported by Li et al. [27], whereas an even lower rate of dry skin was noted in the EF group compared to SF (*p* = 0.08) by Breji et al. [22]. Li et al. reported a low number of adverse events without any differences between MFGM, both for the SF and BF groups. On the other hand, Billeaud et al. (MFGM-P) showed a significantly higher rate of eczema (*p* = 0.001) in infants consuming MFGM-P formula [12]. Other treatment-emergent adverse events did not differ between groups.

**Table 1:** Characteristics of included studies.

Study ID	Study Design and Analysis Method	Population	Sample size + FU (%) at the End of Intervention or Specific Assessment Point	Intervention and Duration	Comparison	Measurement Points	Primary Outcomes	Secondary Outcomes	Results
Zavaleta et al. [24] (Peru) 2011	Double-blind RCT ITT analysis	Healthy children, aged 6 to 11 months old, born at term with a birth weight >2500 g, primarily breast-fed	Inclusion: 550 infants - 277 EF - 273 SF Completed: 91% of infants - 91% EF - 90% SF	*Intervention:* Complementary daily food (40 g/day) with the protein source being the MFGM protein fraction (Lacprodan MFGM-10, Arla Foods Ingredients) *Duration:* 6 months	Complementary daily food (40 g/day) with the protein source being skim milk proteins	(1) Monthly assessment by healthcare professional (2) Twice per week assessment of dietary intake (3) Blood samples collection—beginning and end of intervention	Differences between groups with regard to diarrhea morbidity	(1) Anthropometric evaluation (2) Dietary assessment (3) Hematological and biochemical assessment (hemoglobin, serum ferritin, zinc and folate).	(1) No significant difference in severe diarrhea incidence between groups—reduction of bloody diarrhea incidence in EF group (*p* = 0.025) (2) No differences in biochemical serum parameters between groups (3) Lower Hb level in EF group in 9–10 months of age
Gurnida et al. [25] (Indonesia) 2012	Double-blind RCT PP analysis	Healthy, term infants between 2 and 8 weeks of age with birth weight ≥2.5 kg	*Inclusion*: 110 infants - 35 EF - 35 SF - 40 BF *Completed*: 83% of infants - 83% EF - 86% SF - 80% BF	*Intervention:* Standard formula with added complex milk lipid to increase the ganglioside GD3 (Anmum Infacare, Fonterra) *Duration:* From enrollment (2–8 weeks) until 6 months (24 weeks)	(1) Standard formula (2) Breastfeeding (reference group)	(1) At baseline and at monthly intervals until 6 months old (2) GMDS before and after intervention	Cognitive development using the GMDS	(1)Anthropometric measurements (2) Serum laboratory measurements	(1) Significant increase in scores for Hand and Eye Coordination (*p* = 0.006) and Performance (*p* < 0.001) and also for Total Score (General IQ) (*p* = 0.041) in the EF group but no differences between EF and BF group (2) No significant differences in anthropometric measurements between EF and SF groups (3) Significantly higher serum level of ganglioside G3 in EF compared to SF (*p* < 0.01) with no difference between EF and BF groups
Veereman-Wauters et al. [23] (Belgium, France, the Netherlands) 2012	Double-blind RCT PP analysis	Healthy, pre-school children aged 2.5–6 years old	*Inclusion:* 253 children *Completed:* 72% of children - 67.5% intervention group - 76.4% control group	*Intervention:* 200-mL chocolate formula milk enriched with 500 mg of phospholipids with the addition of 2.5% of MFGM (INPULSE, Büllinger SA) *Duration:* 4 months	200-mL chocolate formula milk	(1) Diaries with daily reports collected every 2 weeks by study coordinator (2) ASEBA assessment at day 120 of study	Number of days with fever, diarrhea, coughing, and/or constipation	(1) Number of doctor visits (2) Medication intake (3) Number of missed schooldays (4) Acceptability of the study product	(1) No difference between groups for diarrhea, constipation, cough, doctor visits, and days of school absence, medication intake (2) The number of days with fever (>38.5 °C) and the number of short (<3 day) febrile periods were significantly (*p* < 0.03) decreased in the intervention group
Billeaud et al. [12] (France, Italy) 2014	Double-blind RCT ITT analysis	Healthy, full-term infants, ≤14 days of age, with a birthweight 2500–4500g	*Inclusion:* 199 infants - 72 EF (MFGM–P) - 70 EF (MFGM–L) - 57 SF *Completed:* 72.3% of infants - 72.2% EF (MFGM–P) - 67.1 EF (MFGM–L) - 78.9% SF	*Interventions:* - Lipid-rich MFGM (Anmum Infacare, Fonterra), - Protein-rich MFGM (Lacprodan MFGM-10, Arla Foods Ingredients) *Duration:* Intervention from 14 (±3) days of age to 4 months (day 112) ≈14 weeks	Standard formula	Baseline visit (0–13 day), day 14 ± 3, 56 ± 5, 84 ± 7, 112 ± 7 plus 3 additional follow-up visits at 6, 9, and 12 months of age in France	Mean weight gain (g/day) from baseline (age 0–13 days) to age 112 days (≈age 4 months), with a non-inferiority margin of −3.0 g/day	(1) Anthropometric measurements (2) Plasma laboratory measurements (3) Immune response to Polio and Hib vaccines	(1) No significant differences in anthropometric measurements ) No differences in plasma phospholipid, cardiolipin, cholesterol, IGF-1, leptin levels (3) Lower C-peptide levels in MFGM-L and P group compared to control formula (*p* = 0.03) (4) Lower mean polio virus type 1 IgG level in the MFGM-P group in Italy (*p* = 0.04)
Poppitt et al. [26] (India) 2014	Double-blind RCT ITT analysis	Healthy infants <2 months of age, gestational age 37–42 weeks, birth weight 2500–4500 g	*Inclusion:* 450 of infants *Completed:* 93% - 94% EF - 91% SF	*Intervention:* High ganglioside complex milk lipid supplement (Anmum Infacare, Fonterra) added to fresh milk *Duration:* For 12 weeks	Control supplement added to fresh milk	Baseline, assessment by fieldworker twice a week (24 visits), after 12-week intervention	Total number of days with RVD during the intervention	(1) Total number of days with diarrhea of any type (ACD) (2) Number of episodes, duration, and severity of RVD and ACD (3) RV load in stool samples at baseline and 12-week	(1) Mean duration that RVD persisted was lower in the EF group (*p* = 0.03) (2) The reported prevalence of major illness during the period of 12 weeks was lower in the EF group (OR 3.5, 95% CI 0.9–20.4, *p* = 0.05)
Timby et al.* [11] (Sweden) 2014	Double-blind RCT ITT analysis	Healthy infants <2 months of age, gestational age 37–42 weeks, birth weight 2500–4500 g	*Inclusion:* 240 infants -80 EF -80 SF -80 BF *Completed (6 months of age):* 92% of infants - 95% EF - 90% SF - 90% BF	*Intervention:* Experimental formula supplemented with protein-rich bovine MFGM (Lacprodan MFGM-10, Arla Foods Ingredients) *Duration:* From enrollment <2 months to 6 months of age, follow-up until 12 months of age	(1) Standard formula (2) Breastfeeding (reference group)	At inclusion, 4, 6, 12 months of age	(1) Cognitive level at 12 month using Bayley-III (2) Weight at 6 months	(1) Anthropometric measurements (2) Plasma inulin	(1) Significantly higher cognitive development in EF group than in SF (*p* = 0.008) but not different from BFR group (2) When groups were divided into BFR compared with formula-fed groups (EF + SF), significant interactions were found between group and time on z-scores for weight (*p* = 0.025) and length (*p* = 0.003) (3) Formula (EF + SF) fed infants had higher insulin plasma concentrations at baseline and 4, 6 months compared to BF group
Timby et al.* [14] (Sweden) 2014	Double-blind RCT ITT analysis	Healthy infants <2 months of age, gestational age 37–42 weeks, birth weight 2500–4500 g	*Inclusion:* 240 infants -80 EF -80 SF -80 BF *Analysis at:* - 4 months of age: 92.5% - 6 months of age: 92% - 12 months of age: 92%	*Intervention:* Experimental formula supplemented with protein-rich bovine MFGM (Lacprodan MFGM-10, Arla Foods Ingredients) *Duration:* From enrollment <2 months to 6 months of age, follow-up until 12 months of age	(1) Standard formula (2) Breastfeeding (reference group)	At inclusion, 4, 6, 12 months of age	Serum lipids, adipokines, homocysteine, inflammatory biomarkers, and blood pressure	NA	(1) Until 6 mo, the EF group had higher total serum cholesterol concentration than the SF group, reaching the level of the BF group (2) Blood pressure did not differ significantly between groups
Timby et al.* [15] (Sweden) 2015	Double-blind RCT ITT analysis	Healthy infants <2 months of age, gestational age 37–42 weeks, birth weight 2500–4500 g	*Inclusion:* 240 infants - 80 EF - 80 SF - 80 BF *Analysis at:* - 6 months of age: 93% - 12 months of age: 75%	*Intervention:* Experimental formula supplemented with protein-rich bovine MFGM (Lacprodan MFGM-10, Arla Foods Ingredients) *Duration:* From enrollment <2 months to 6 months of age, follow-up until 12 months of age	(1) Standard formula (2) Breastfeeding (reference group)	(1) Diaries filled by parent until 6 and 12 months of age (2) IgG level to pneumococci at 12 month of age	(1) Incidence of infections throughout 12 months (2) Infection-related medication intake (2) Serum concentration of IgG to pneumococci	NA	(1) Fewer acute otitis media episodes in EF compared to the SF group (*p* = 0.034) (2) The incidence (*p* = 0.021) and longitudinal prevalence (*p* = 0.012) of antipyretic use were significantly lower in the EF group (3) EF group had lower s-IgG concentrations for *S. Pneumoniae* serotypes 1, 5, and 14 compared with the SF group
Timby et al.* [16] (Sweden) 2017	Double-blind RCT Analysis of randomly selected infants	Healthy infants <2 months of age, gestational age 37–42 weeks, birth weight 2500–4500 g	*Inclusion:* 240 infants - 80 EF - 80 SF - 80 BF *Analysis at:* - 4 months of age 52% - 12 months of age: 69%	*Intervention:* Experimental formula supplemented with protein-rich bovine MFGM (Lacprodan MFGM-10, Arla Foods Ingredients) *Duration:* From enrollment <2 months to 6 months of age, follow-up until 12 months of age	(1) Standard formula (2) Breastfeeding (reference group)	Control visits at 4 and 12 months of age	Oral microbiota composition	NA	(1) The species richness did not differ between the EF and SF group at 4 or 12 months of age (2) BF group had significantly lower species richness in oral microbiota than the formula-fed groups at 4 and 12 month of age
Grip et al.* [17] (Sweden) 2018	Double-blind RCT Analysis of the serum of randomly selected infants from each group	Healthy infants <2 months of age, gestational age 37–42 weeks, birth weight 2500–4500 g	*Inclusion:* 240 infants - 80 EF - 80 SF - 80 BF Analysis at: - 4 month of age: 90 randomly chosen infants - 6 months of age: 89% of infants - 12 months of age: 90 randomly chosen infants	*Intervention:* Experimental formula supplemented with protein-rich bovine MFGM (Lacprodan MFGM-10, Arla Foods Ingredients) *Duration:* From enrollment: <2 months to 6 months of age, follow-up until 12 months of age	(1) Standard formula (2) Breastfeeding (reference group)	Blood samples were collected at 4, 6, and 12 months of age and were obtained >2 h after the latest meal in randomly selected infants	(1) Plasma lipidome and erythrocyte membrane lipidome (6 months) (2) Serum lipidome (4 and 12 months)	NA	There were significant differences in the serum/plasma lipidome at 4 and 6 months of age in infants fed the EF compared to infants fed SF. This separation was also detected in erythrocyte membranes at 6 months, but it did not remain in sera collected at 12 months of age, 6 months after the end of the intervention
He X. et al.* [18] (Sweden) 2019	Double-blind RCT Analysis of the serum and plasma of randomly selected infants from each group	Healthy infants <2 months of age, gestational age 37–42 weeks, birth weight 2500–4500 g	Longitudinal assessment: 90 randomly selected infants out of 240 Cross-sectional assessment: 212 infants out of 240	*Intervention:* Experimental formula supplemented with protein-rich bovine MFGM (Lacprodan MFGM-10, Arla Foods Ingredients) *Duration:* From enrollment: <2 months to 6 months of age, follow-up until 12 months of age	(1) Standard formula (2) Breastfeeding (reference group)	At enrollment, 4, 6, 12 months of age: serum for longitudinal assessment 6 month of age- plasma for cross-sectional assessment	Metabolome	NA	(1) Formula-fed infants had higher levels of amino acid catabolism by-products and a low efficiency of amino acid clearance (preference for protein metabolism) (2) BF infants had higher levels of fatty acid oxidation products (preference for fat metabolism)
He X. et al.* [19] (Sweden) 2019	Double-blind RCT Analysis of fecal microbiome and metabolome of randomly selected infants from each group	Healthy infants <2 months of age, gestational age 37–42 weeks, birth weight 2500–4500 g	Randomly selected subset of 90 infants (15 females and 15 males from each group: BF, SF, and BF) out of 240 infants	*Intervention:* Experimental formula supplemented with protein-rich bovine MFGM (Lacprodan MFGM-10, Arla Foods Ingredients) *Duration:* From enrollment: <2 months to 6 months of age, follow-up until 12 months of age	(1) Standard formula (2) Breastfeeding (reference group)	At enrollment, 4, 6, 12 months of age	Fecal microbiome and metabolome	NA	(1) Fecal metabolome of EF-fed infants showed a significant reduction of lactate, succinate, amino acids, and their derivatives compared to SF-fed infants (2) Introduction of weaning food with either human milk or infant formula reduces the distinct characteristics of breastfed or formula-fed infant fecal microbiome and metabolome profiles
Breij et al. [22] (Belgium, France, the Netherlands, Singapore) 2019	Double-blind RCT PP analysis	Healthy, term infants (gestational age 37–42), postnatal age ≤35 d, at birth weight between 10th and 90th percentiles	*Inclusion:* 313 infants - 115 EF - 108 SF - 88 BF *Completed:* 76% of infants - 76% EF - 75% SF - 78% BF	*Intervention:* Experimental formula comprising large, milk phospholipid-coated dairy lipid droplets *Duration:* From enrollment: ≤35 days of age to 17 weeks of age	(1) Standard formula with small lipid droplets containing vegetable oils (2) Breastfeeding (reference group)	Baseline visit (≤35 day of age), 5, 8, 13, 17 weeks of age	Daily weight gain until 17 weeks of age	(1) Anthropometric measurements (2) Formula intake (3) Tolerance (4) Stool characteristics (5) Fat-soluble vitamins in plasma (6) Adverse events	(1) No relevant differences were observed in growth in all groups (2) Lower percentage of subjects with the atopic eczema in the EF compared with the SF group (*p* = 0.03) (3) No differences in plasma A and E vitamins concentration (*p* = 0.423, *p* = 0.62 respectively)
Li X et al. [13] (China) 2019	Double-blind RCT ITT analysis	Healthy infants aged 21 ± 7 days with gestational age of 37–42 weeks at birth, birth weight >2500 g and <4000 g	*Inclusion:* 789 infants: - 192 EF - 195 PF - 194 SF - 208 BF *Completed:* 87% of infants - 87% EF - 88% PF - 86% SF - 86% BF	*Intervention:* (1) Experimental formula supplemented with protein-rich bovine MFGM (Lacprodan MFGM-10, Arla Foods Ingredients) (2) Milk formula supplemented with *L. paracasei* strain F19 (Chr. Hansen) *Duration:* From enrollment (21 ± 7 days) until 4 months of age	(1) Standard formula (2) Breastfeeding (reference group)	At baseline and at 1, 2, 3, 4, 5, 6, 9, and 12 month of age	Incidence of infections, diarrhea episodes and days with fever	(1) Anthropometric measurements (2) Safety and tolerability	(1) Mean weight for the breastfed group was significantly higher than for the F19 group until age 2 months (*p* = 0.015 and 0.028 at 1 and 2 months, respectively) and for the SF and MFGM groups until age 4 months (all *p* < 0.041). After these ages’ infants showed no significant differences (2) Compared to the breastfed group, the SF infants had significantly more fever episodes (*p* = 0.021) and days with fever (*p* = 0.036), but not episodes of diarrhoea (3) Neither the MFGM nor the F19 groups had significantly more episodes of fever or number of days with fever than the breastfed group
Li F et al. [27] (China) 2019	Double-blind RCT ITT analysis	Healthy, full-term infants, 10–14 days of age at randomization, exclusively formula fed for at least 3 days before randomization, birth weight of 2500–4000 g, singleton birth	*Inclusion:* 451 infants - 223 EF - 228 SF *Completed:* 65% - 65% EF - 65% SF	*Intervention:* Experimental formula supplemented with protein-rich bovine MFGM (Lacprodan MFGM-10, Arla Foods Ingredients) and bovine lactoferrin *Duration:* From 10–14 day of age until 365 day of age	Standard formula	(1) Bayley-III at day 365 and 545, (2) Anthropometric measurements at day 30, 42, 60, 90, and 120	Cognitive level at day 365 (age of infant)	(1) Anthropometric measurements (2) Medically confirmed adverse events (3) Cognitive level at day 545 (4) Other instruments for child’s development at various time points	(1) Higher scores in cognitive (*p* < 0.01), motor (*p* < 0.01), and language functions (*p* < 0.01) in MFGM group at day 365 (2) No group differences were detected (adjusted or unadjusted) in any Bayley-III domain in participants tested at day 545 (3) Lower rate of respiratory and gastrointestinal AEs in MFGM + lactoferrin group; no differences in eczema occurrence
Nieto-Ruiz et al. [20] (Spain) 2019	Double-blind RCT PP analysis	Healthy 0–2-month-old full-term infants w/adequate birth weight for gestational age, normal Apgar score	*Inclusion:* 220 infants: - 85 EF - 85 SF - 50 BF *Completed:* 64% of infants - 66% EF - 56% SF - 76% BF	*Intervention:* EF containing LCPUFAs AA, DHA, MFGM, symbiotics, gangliosides, nucleotides, and sialic acid *Duration:* From 0–2 month up to 18 months of age	(1) Standard formula (2) Breastfeeding (reference group)	(1) Baseline, 2, 3, 4, 6, 12, 18 months of age for anthropometric evaluation (2) Neurological development assessment at 2, 3, 4, and 12 months of age	(1) Weight, length, and subsequent body mass index (BMI) (2) Neurological Development (3) Visual Function	NA	(1) All infants presented an adequate neurological development up to 4 months of life, and no statistically significant differences were found between all the groups at any point in time
Nieto-Ruiz et al. [21] (Spain) 2020z	Double-blind RCT PP analysis	Healthy 0–2-month-old full-term infants w/adequate birth weight for gestational age, normal Apgar score	*Inclusion:* 220 infants: - 85 EF - 85 SF - 50 BF *Completed:* 55% of infants - 50% EF - 54% SF - 66% BF	*Intervention:* EF containing LCPUFAs AA, DHA, MFGM, symbiotics, gangliosides, nucleotides and sialic acid *Duration:* From 0–2 months old up to 18 months of age	(1) Standard formula (2) Breastfeeding (reference group)	At 4 years of age	Assessments of oral language development in kindergarten children	NA	(1) Children who received EF seemed to show higher scores in the use of language (*p* = 0.033) and oral spontaneous expression (*p* = 0.024) than children who received SF (2) SF children presented lower scores in language content (*p* = 0.026) and total score of PLON-R test (*p* = 0.029) compared to BF children in an unadjusted model. After adjustment for selected confounding variables differences disappeared

* Timby—one cohort; Nieto–Ruiz—one cohort; -RCT—Randomized controlled trial; -ITT—Intention to treat analysis; -PP—Per protocol analysis; -FU—Follow-up strength; -wks—Weeks; -EF—Experimental formula; -SF—Standard formula; -DHA—Docosahexaenoic acid; -AA—Arachidonic acid; -PF—Probiotic containing formula;-BF—Breastfeeding; -RV—Rotavirusk; -RVD—Rotavirus diarrhea; -ACD—Any type diarrhea; -Bayley-III—Bayley Scales of Infant and Toddler Development; -GDMS—Griffiths Mental Development Scale; -ASEBA—Achenbach System of Empirically Based Assessment; -LCPUFAs—Long-chain polyunsaturated fatty acids.

## 4. Discussion

The deepening understanding of complex communication between distinct organs, such as gut–brain and gut–lung axes, has expanded the significance of the breast milk to support the optimal cognitive and physical development of a child [28,29]. Therefore, unceasing efforts are being taken to create an infant formula mimicking the composition of the breast milk as closely as possible. It has been suspected that MFGM may be responsible for some of the advantageous properties of breastfeeding. Recently, a novel formula milk enriched with MFGM, naturally occurring in the maternal milk, was placed on the market. Numerous questions have been risen about the tangible evidence and whether to recommend it in the clinical setting. This infant milk is advertised as a product stimulating brain development, along with strengthening the immune system.

The beneficial role of MFGM has already been explored in animals and adults. Studies on rodent models have proven the impact of MFGM on infection, inflammation, brain composition, and gut barrier integrity, as well as intestinal development [30,31,32,33,34]. Interestingly, pups fed control formula demonstrated delayed intestinal growth, while the MFGM supplementation normalized intestinal crypt depths, epithelial cell proliferation, make-up of intestinal epithelial cell subsets, and resembled the make-up of intestinal microbes of mother’s milk-fed rats at the phylum level [34]. This finding may be specifically important for premature infants born with immature gastrointestinal tract, resulting in their higher risk of infections and a life-threatening Necrotizing Enterocolitis (NEC) [35]. Moreover, this study showed that the addition of MFGM also afforded significant protection to *Clostridium difficile* toxins, which may be an attractive feature as there is growing evidence that *C. difficile* is associated with diarrhea and prolonged hospitalization in younger children [34,36,37].

The results of our primary outcome meta-analyses have successfully confirmed that all the MFGM supplemented formulas have shown the non-inferiority when it comes to growth and weight parameters in children, or it even represented the results that were more similar to the breastfed groups. These conclusions had been anticipated beforehand, as formula feeding has been associated with infant’s increased weight gain velocity compared to breastfeeding [38,39]. Another key thing to remember is the very good safety profile of these formulas. Unfortunately, there is a small amount of evidence about the reported promising effects, which are fewer episodes of fever and lower need to use antipyretics, lower incidence of acute otitis media, reduction in episodes or duration of diarrhea, and improved cognitive development, which does not permit us to draw any firm conclusions about the effectiveness of MFGM supplementation in infants and children.

Yet, several issues have arisen during the assessment. Importantly, our findings revealed a limited number of pertinent clinical trials and a considerable heterogeneity among them. Firstly, although the composition of the milk formulas used was comparable, several different MFGM supplement products were applied in these trials. Due to the addition of other supplements enriching concept formulas along with MFGM, the results of two trials could not be analyzed equally [20,21,27]. The long-chain polyunsaturated fatty acids (LCPUFA) supplementation during pregnancy and/or lactation might be associated with a beneficial impact on child’s neurodevelopment [40,41]. Moreover, current evidence suggests that prophylactic use of lactoferrin, a protein found in cow and human milk, in preterm infants could significantly reduce the incidence of NEC [42]. Secondly, intervention duration and length of follow-up differed significantly between the studies. Thirdly, the experimental outcomes, such as an incidence of infection or makeup of the oral microbiome, were often inspected in individual trials [13,15,16]. Furthermore, cognitive development was evaluated with various measurement scales. Timby et al. used the Bayley-III score, while Li et al. employed the GMDS at different time points, making the outcomes harder to compare [11,13].

Our systematic review has several strengths. A key asset is a brief presentation, with a quantitative and qualitative synthesis of data, of the novel formula enrichment on the market. The thorough search of the most extensive databases complemented with seeking the information from conference abstracts, contacting study authors and experts in the field makes it the most comprehensive summary of all experimental data about the use of MFGM in children up to date. Furthermore, this review provides an overview of currently ongoing trials registered at the ClinicalTrials, the EU Clinical Trials Register, and the Australian New Zealand Clinical Trials Registry (ANZCTR) (Table 2). Finally, when there is enough evidence, the mentioned supplementation could become an easy-to-use and cost-efficient add-on approach for many parents to support a child’s cognitive development and lower the prevalence of acute otitis media or fever episodes.

We are also aware of the limitations of our study. Trials presenting positive results are more eagerly published than those with negative results; therefore, this might be a source of positive-results bias. We could fully compare and contrast the findings of all included trials, as some of them assessed several supplements simultaneously.

## 5. Conclusions

Our review is the first systematic attempt to provide a valid answer to the question as to whether the present evidence is convincing enough to commend milk formulas enriched with MFGM. The currently available data signaled beneficial effects and a good safety profile, requiring future research with well-designed randomized double-blind placebo-controlled trials measuring the outcomes in a more unified way.

## Figures and Tables

**Table 2:** Ongoing clinical trials on MFGM supplementation in children.

Protocol ID, Trial Registry	Location	Title	Recruitment Status	Estimated Completion Date
NCT04508257, ClinicalTrials.gov	China, Zhejiang	The Effect of a New Infant Formula on Growth and Cognition in Healthy Term Infants	Currently Recruiting	28 February 2023
ACTRN12620000552987, ANZCTR.org.au	Australia	Infant nutrition with milk fat globule membrane for infant cognition in early life	Not Yet Recruiting/Currently Recruiting	23 March 2024
NCT02626143, ClinicalTrials.gov, BMC Pediatrics [43]	Chile	Effect of feeding mode on infant growth and cognitive function: study protocol of the Chilean infant Nutrition randomized controlled Trial (ChiNuT)	Recruitment Completed	NA

NA—Not Applicable.

## Data Availability

No new data were created or analyzed in this study. Data sharing is not applicable to this article.

## Supplementary Materials

The following are available online at https://www.mdpi.com/2072-6643/13/3/714/s1, Figure S1: Risk of bias assessment visualized using ROBVIS tool, Table S1: Pubmed search strategy.
