# Supplementary material for: Milk Fat Globule Membrane Supplementation in Children: Systematic Review with Meta-Analysis
Source: Nutrients. 2021 Feb 24;13(3):714. doi: 10.3390/nu13030714 (PMC7996302; doi:10.3390/nu13030714)
Supplement: Supplementary file 1 [file nutrients-13-00714-s001.pdf]

*Supplementary Materials*

# Milk Fat Globule Membrane supplementation in children: Systematic Review with Meta-analysis

**Dominika Ambrożej [1,2], Karolina Dumycz [1,2], Piotr Dziechciarz [3] and Marek Ruszczyński [3,*]**

1 Department of Pediatric Pneumonology and Allergy, Medical University of Warsaw, Poland; alergologia@spdsk.edu.pl
2 Doctoral School, Medical University of Warsaw, Poland; szkoladoktorska@wum.edu.pl
3 Department of Pediatrics, Medical University of Warsaw, Poland; pediatria@wum.edu.pl
* Correspondence: marek.ruszczynski@wum.edu.pl

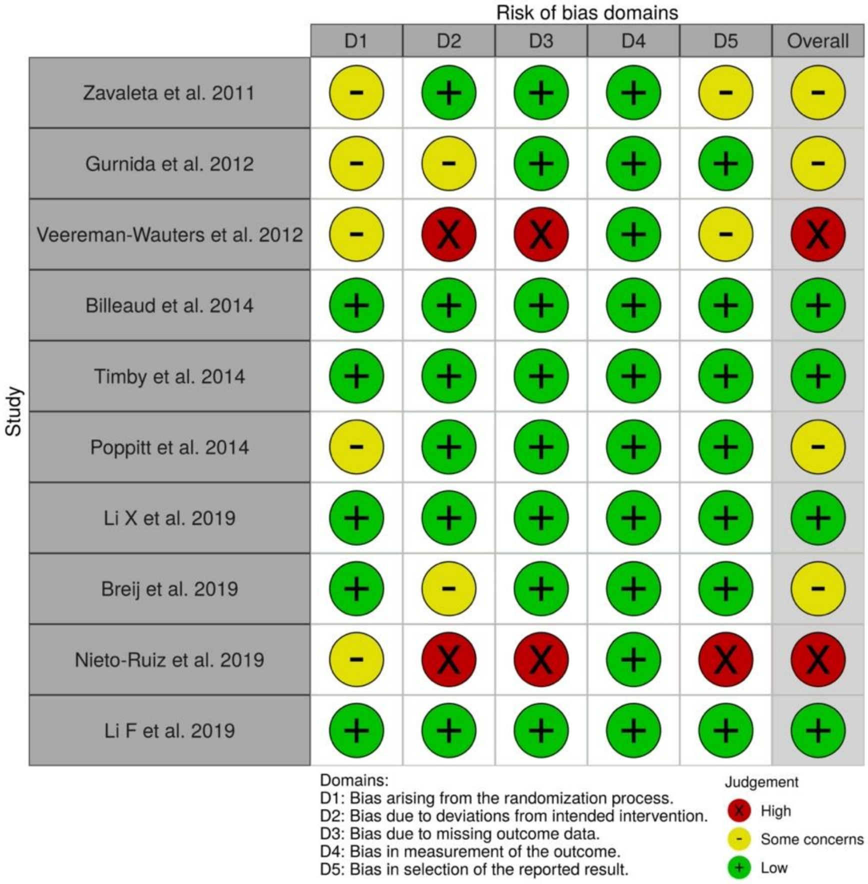


**Figure S1.** Risk of bias assessment visualized using ROBVIS tool.

**Table S1.** Pubmed search strategy.

| |
|---|
| #1 randomized controlled trial [pt] |
| #2 controlled clinical trial [pt] |
| #3 randomized [tiab] |
| #4 placebo [tiab] |
| #5 drug therapy [sh] |
| #6 randomly [tiab] |
| #7 trial [tiab] |
| #8 groups [tiab] |
| #9 #1 OR #2 OR #3 OR #4 OR #5 OR #6 OR #7 OR #8 |
| #10 animals [mh] NOT humans [mh] |
| #11 #9 NOT #10 |
| #12 (MFGM*) OR (milk AND fat AND globule) OR (milk AND fat AND globule AND (membrane OR membranes)) OR (glycolipids AND ((MFGM*) OR (milk AND fat AND globule))) OR (glycoproteins AND ((MFGM*) OR (milk AND fat AND globule))) OR (complex AND milk AND lipid) |
| #13 #11 AND #12 |
